# A Scientometric Review of Soft Robotics: Intellectual Structures and Emerging Trends Analysis (2010–2021)

**DOI:** 10.3389/frobt.2022.868682

**Published:** 2022-05-05

**Authors:** Yitong Zhou, Haonan Li

**Affiliations:** Shien-Ming Wu School of Intelligent Engineering, South China University of Technology, Guangzhou, China

**Keywords:** soft robotics, bio-inspired robots, scientometrics, co-citation analysis, CiteSpace

## Abstract

Within the last decade, soft robotics has attracted an increasing attention from both academia and industry. Although multiple literature reviews of the whole soft robotics field have been conducted, there still appears to be a lack of systematic investigation of the intellectual structure and evolution of this field considering the increasing amount of publications. This paper conducts a scientometric review of the progressively synthesized network derived from 10,504 bibliographic records using a topic search on soft robotics from 2010 to 2021 based on the Web of Science (WoS) core database. The results are presented from both the general data analysis of included papers (e.g., relevant journals, citation, h-index, year, institution, country, disciplines) and the specific data analysis corresponding to main disciplines and topics, and more importantly, emerging trends. CiteSpace, a data visualization software, which can construct the co-citation network maps and provide citation bursts, is used to explore the intellectual structures and emerging trends of the soft robotics field. In addition, this paper offers a demonstration of an effective analytical method for evaluating enormous publication citation and co-citation data. Findings of this review can be used as a reference for future research in soft robotics and relevant topics.

## 1 Introduction

Soft robotics is a rapidly growing and fast-moving interdisciplinary field of study; within the last decade the field has expanded with a dedicated journal and conference, worldwide open-access resources, and collaboration platforms. Compared to conventional rigid robots, soft devices are more adept at navigating uneven terrains, resilient to perturbations, flexible to changing task settings, and safer to interact with humans. Demonstrated applications include rehabilitation devices (Chu and Patterson, 2018), untethered robots moving through unpredictable terrains (Tolley et al., 2014), manipulators (Dou et al., 2021; Zhou et al., 2020; Zhou et al., 2021a), various types of soft grippers (Shintake et al., 2018; Zhou et al., 2022; Zhou et al., 2021b) etc.

The efforts to create soft robots started long before the emergence of the professional term “soft robot” such as McKibben actuators developed in the 1950s. However, the field only started to gain momentum around 2008 indicated by a brief history of soft robots in (Bao et al., 2018). According to our literature survey, Trivedi et al. (2008) were the first to review soft robotics field in 2008, where soft robots were firstly differentiated from hard, hyper-redundant robots when the field was still in infancy. As soft robotics becomes gradually recognized by the robotics community, a number of review articles have been published to summarize the achievements, analyze the techniques, and discuss the challenges and prospects for the future. Chu et al. investigated soft robotic gloves for hand rehabilitation and established a framework to compare different design aspects (Chu and Patterson, 2018). Amjadi et al. (2016) studied flexible strain sensors used in wearable devices. Polygerinos et al. (2017) focused on fluid-driven intrinsically soft devices. Rich et al. (2018) reviewed functional untethered soft robotics and focused on actuation, sensing, and integration methods. Shintake et al. (2018) presented a soft gripper review covering different material sets, physical principles, and device architectures. Gul et al. (2018) and Wallin et al. (2018) both summarized state-of-the-art 3D printing methods for soft robotics. Stano et al. (Stano and Percoco, 2021) gave a review of the current use of additive manufacturing (AM) methods for the manufacturing of soft robots. Cianchetti et al. (2018) reviewed biomedical applications of soft robotics such as soft tools for surgery, diagnosis and drug delivery, wearable and assistive devices. Manti et al. (2016) investigated the stiffening approaches through direct activation of soft actuation technologies. And the list goes on. While the above reviews demonstrated the evolution and characteristics of soft robots in specific domains, there were also reviews that investigate soft robots from a general perspective including multiple earlier reviews (Trivedi et al., 2008; Kim et al., 2013; Majidi, 2014; Rus and Tolley, 2015) and recent ones (Lee et al., 2017; Whitesides, 2018). These reviews provide detailed overviews of soft robotics and prospects for the future over time. As research in soft robotics areas advances rapidly, it is critical to keep abreast of emerging trends and critical turns of the development of the collective knowledge. However, the body of the relevant literature grows rapidly (over 1,700 articles each year), making reviews of the whole field much harder.

Scientometrics is a branch of informatics that quantitatively analyzes patterns in scientific literature in order to understand emerging trends and the knowledge structure of a research. CiteSpace, a science mapping tool designed for scientometrics analysis, takes scientific publications as an input and generate interactive visual representations of complex structures for statistical analysis and interactive visual exploration. It has been widely used for domain analysis and visualization for large volumes of scientific literature in a variety of disciplines, such as medicine (Chen et al., 2012), smart cities (Zheng et al., 2020), information science (Yu et al., 2017), and sustainable development (Olawumi and Chan, 2018). Yet, to our knowledge, this is the first scientometric analysis to assess the general soft robotics field and analyze emerging trends based on a data-driven approach.

The aim of this study is to reveal the inner knowledge structure and citation landscape of soft robotics publications from a general perspective by revealing the following aspects: (1) the development map; (2) the main contributors, countries, institutions, authors, and cooperative relationships; (3) the main research disciplines and topics; and (4) emerging trends in soft robotics. We review all research areas in the soft robotics field from 2010 to 11 July 2021, which covers a wide spectrum of research areas. CiteSpace is used to visualize knowledge structures, analyze specific leading influence articles, and summarize emerging trends. The rest of this paper is organised as follows. Section 2 presents the systematic literature review method and the searching principles. Section 3 illustrates and discusses the obtained characteristic outputs for the number of publications, countries, organizations, journals, subject areas etc. Section 4 investigates the intellectual structures of soft robotics field. Section 5 proposes research trends for the future by analyzing emerging intensity. Section 6 concludes this paper.

## 2 Publication Selection Procedure

**FIGURE 1:**
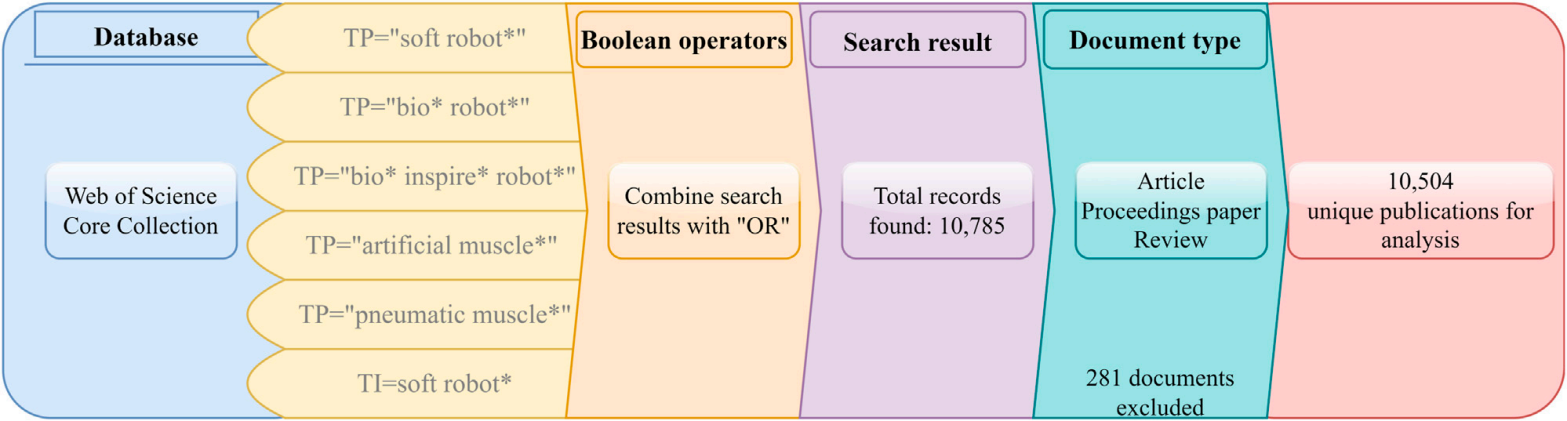
The flowchart that reports the publication selection procedure (TP, topic; TI, title).

The analysis is based on the publications related to “soft robot” published from 2010 to 2021. The flowchart that reports the selection procedure is shown in Figure 1. Literature were retrieved in the Web of Science (WoS) Core Collection on 11 July 2021 with a combination of: (1) searching by topic: “soft robot*,” “bio* robot,” “bio* inspire* robot*,” “artificial muscle*,” “pneumatic muscle*”; and (2) searching by title: soft robot*. Here, searching by topic includes searching by title, abstract, author keywords, and KeyWords Plus. KeyWords Plus (Clarivate, 2018) are words or phrases that frequently appear in the titles of an publication’s references, but do not appear in the title of the publication itself, enhancing the power of cited-reference searching. The quotation marks in (1) retrieve the exact phrase inside. For example, the query “soft robot*” finds records containing the exact phrase soft robot, soft robots, soft robotics etc. It does not match robot soft, soft flying robot, or soft pneumatic robot. This helps exclude irrelevant articles and results in more consistent publications. To generate an appropriate search space, we determine the search strategy based on two main aspects. Firstly, based on multiple review articles over time (Trivedi et al., 2008; Kim et al., 2013; Rus and Tolley, 2015; Bao et al., 2018), we collect a few possible searching phrases, which are then reduced if including some phrases would introduce too many irrelevant articles into our database. For example, “soft robot*” is reasonable, but adding “soft material*” to (1) will lead to many articles in the field of pure materials or chemistry, which is not desired. Secondly, we include phrases that indicate soft robots but have been independently used over time. For example, the choice of “pneumatic muscle” or “artificial muscle.” The term “soft robot” was not commonly used until around a decade ago, but the idea of soft robots may date back to Mickibben pneumatic artificial muscles in the 1950s. In recent years, some of the articles have kept using the terms “pneumatic muscle” or “artificial muscle” alone, which belong to soft robotics by the current definition. In addition to the proposed phrases, we tried to explore other phrases using searching by topic, such as adding popular applications of soft robots, including “soft gripper*,” “soft crawler*,” “soft walker*,” “soft manipulator*” and “soft tentacle*,” but found they barely expanded the search results and hence decided not to include them. This may indicate that the proposed search phrases indeed envelop the appropriate search space for soft robotics, and that our proposed search strategy is reasonable. We combine all search results with an “OR” operation and a total of 10,785 documents were obtained. Then, we restrict the document type as article, proceedings paper, and review. As a result, 10,504 publications in the field of soft robotics were collected. In the rest of this paper, we will use articles interchangeably with publications for convenience.

## 3 Characteristics of Article Outputs

The data source for this study include 10,504 publications found in the Web of Science dating between 2010 and 2021 (until 11 July 2021; to be more precise).

### 3.1 The Number of Publications

**FIGURE 2:**
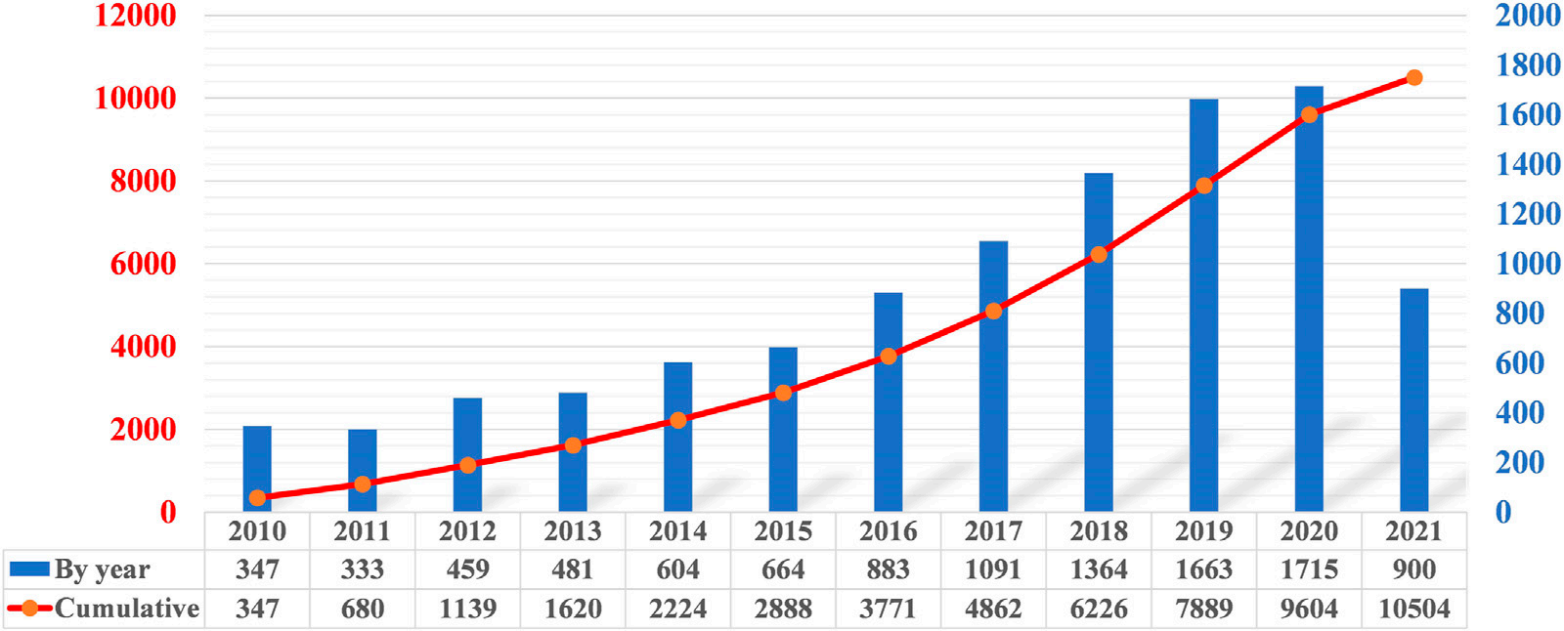
The number of publications related to soft robotics by year and cumulatively.

The number of soft robotics publications in 2010 and 2011 average 340 per year and are fairly close, as shown in Figure 2. Then, the yearly publications increase significantly by 38% to 459 in 2012. The increasing trend continues with yearly increments of publications from dozens to a few hundreds during 2012–2015. Starting 2016, the rising rate of yearly publications has been relatively stable, with high percentages of 33%, 24%, 25%, and 22% in 2016, 2017, 2018, and 2019, respectively. It is worth noting that the yearly publications are more than 1,000 for the first time in 2017. The active interests and intensive efforts from robotics community in the past few years has led to the significant progress of this field, which is indicated by the enormous increase in the number of publications. Since 2020, the yearly publications increase with a lower growth rate. Compared to 1715 publications in 2020, 900 publications have been contributed in the first 7 months this year, indicating that the growth has started to slow down.

### 3.2 Contribution of Leading Countries

In total, 101 countries or regions contribute to our final publication collection. Table 1 lists the top 20 most productive countries or regions in soft robotics field ranked by the number of total articles (TA) during the last 12 years. It is observed that China is the most productive country with a total publication of 2966, followed closely by the United States (2911), then Japan (1048). However, in terms of average citations per publication (ACPP), the top three countries/regions are the United States (28.82), the Netherlands (28.55), and Belgium (26.36), indicating stronger impact per publication. It is worth noting that, despite being the most productive country, China’s ACPP is relatively low. The situation is similar for Japan. It is unclear whether it is due to research quality itself, language bias, or joining this field late. Another observation is that a large number of publications are international-collaborated articles (28.2%) for the top 20 country/region’s publications, indicating soft robotics has attracted worldwide researchers to work together. In addition, the top four countries also have the largest number of collaborative countries. It is also noted that ACPP is generally higher for international articles, implying that collaborations may have facilitated higher-quality research, hence greater impacts. It may also explain why the Netherlands and Belgium have relatively high ACPP, as their international-collaborated articles account for 65% and 61% of their total publications.

**TABLE 1:** The top 20 most productive countries/regions in soft robotics field during 2010–2021.

Rank	Country/Region	TA	TC	ACPP	H-index	*n* CC	IC				NIC			
							TA	TC	ACPP	SP	TA	TC	ACPP	SP
1	China	2966	41601	14.03	86	45	929	19347	20.83	0.31	2037	22254	10.92	0.69
2	United States	2911	83887	28.82	129	56	972	30669	31.55	0.33	1939	53229	27.45	0.67
3	Japan	1048	10888	10.39	46	46	277	4819	17.40	0.26	771	6070	7.87	0.74
4	United Kingdom	689	9838	14.28	42	49	404	7212	17.85	0.59	285	2626	9.21	0.41
5	South Korea	600	14547	24.25	53	32	179	8437	47.13	0.30	421	6112	14.52	0.70
6	Italy	591	13929	23.57	51	42	287	8044	28.03	0.49	304	5886	19.36	0.51
7	Germany	553	10545	19.07	48	48	289	7575	26.21	0.52	264	2973	11.26	0.48
8	Singapore	365	7054	19.33	42	27	207	4307	20.81	0.57	158	2749	17.40	0.43
9	France	315	5177	16.43	35	40	188	3244	17.26	0.60	127	1933	15.22	0.40
10	Switzerland	309	5909	19.12	41	32	173	2742	15.85	0.56	136	3167	23.29	0.44
11	Australia	306	6560	21.44	39	29	158	4368	27.65	0.52	148	2193	14.82	0.48
12	Canada	243	5392	22.19	34	31	138	3535	25.62	0.57	105	1858	17.70	0.43
13	India	197	1484	7.53	19	30	63	662	10.51	0.32	134	823	6.14	0.68
14	Spain	191	3485	18.25	31	38	97	1981	20.42	0.51	94	1504	16.00	0.49
15	Netherlands	171	4882	28.55	30	36	112	3737	33.37	0.65	59	1148	19.46	0.35
16	New Zealand	157	2372	15.11	27	15	65	855	13.15	0.41	92	1517	16.49	0.59
17	Taiwan	109	1027	9.42	18	18	32	451	14.09	0.29	77	576	7.48	0.71
18	Iran	105	841	8.01	15	15	38	462	12.16	0.36	67	380	5.67	0.64
19	Turkey	99	1395	14.09	16	21	46	1136	24.70	0.46	53	259	4.89	0.54
20	Belgium	96	2531	26.36	22	23	59	1668	28.27	0.61	37	863	23.32	0.39

TA, total articles; TC, total citations; ACPP, average citations per publication; nCC, number of collaborative countries; IC, international collaboration; NIC, not international collaboration; SP, share of publications for IC or NIC in total articles.

### 3.3 Contribution of Leading Institutions

Table 2 lists the most productive 20 institutions in the soft robotics field. Apparently, most of them are from the top 10 most productive countries and China and the United States account for seven and four institutions, respectively. Chinese Academy of Sciences leads the list with the most publications followed by University of California System and Harvard University. In terms of ACPP, institutions from the United States lead the list with Harvard University (74.14) and Massachusetts Institute of Technology (59.81). Apparently these top institutions have taken a prominent role in developing and promoting soft robotics. Except for institutions in the United States, Scuola Superiore Sant Anna (36.09), University of Wollongong (36.09), and Seoul National University (27.96) have the highest ACPP.

**TABLE 2:** The Top 20 Most productive institutions in soft robotics field during 2010–2021.

Rank	Institution	TA	TPR%	TC	ACPP	H-Index	Country/Region
1	Chinese Academy of Sciences	420	3.998	8960	21.33	51	China
2	University of California System	276	2.627	10106	36.62	43	United States
3	Harvard University	256	2.437	18981	74.14	64	United States
4	National University of Singapore	224	2.132	3669	16.38	32	Singapore
5	Istituto Italiano Di Tecnologia	203	1.932	5121	25.23	32	Italy
6	Massachusetts Institute of Technology	197	1.875	11783	59.81	52	United States
7	Tsinghua University	176	1.675	3850	21.88	32	China
8	Harbin Institute of Technology	169	1.609	1640	9.7	21	China
9	Zhejiang University	169	1.609	2146	12.7	22	China
10	Centre National De La Recherche Scientifique	166	1.580	2647	15.95	28	France
11	Scuola Superiore Sant Anna	161	1.532	5811	36.09	32	Italy
12	Xi An Jiaotong University	155	1.475	2443	15.76	26	China
13	University of Wollongong	152	1.447	4872	32.05	34	Australia
14	Shanghai Jiao Tong University	145	1.380	1843	12.71	23	China
15	Beihang University	139	1.323	2751	19.79	26	China
16	Ecole Polytechnique Federale De Lausanne	138	1.314	3150	22.83	28	Switzerland
17	University of Auckland	138	1.314	2309	16.73	27	New Zealand
18	Seoul National University	137	1.304	3830	27.96	33	South Korea
19	University of Bristol	137	1.304	1414	10.32	20	United Kingdom
20	University of Texas System	137	1.304	5252	38.34	36	United States

TPR%, the percentage of articles of journals in total publication.

### 3.4 Main Subject Areas

**FIGURE 3:**
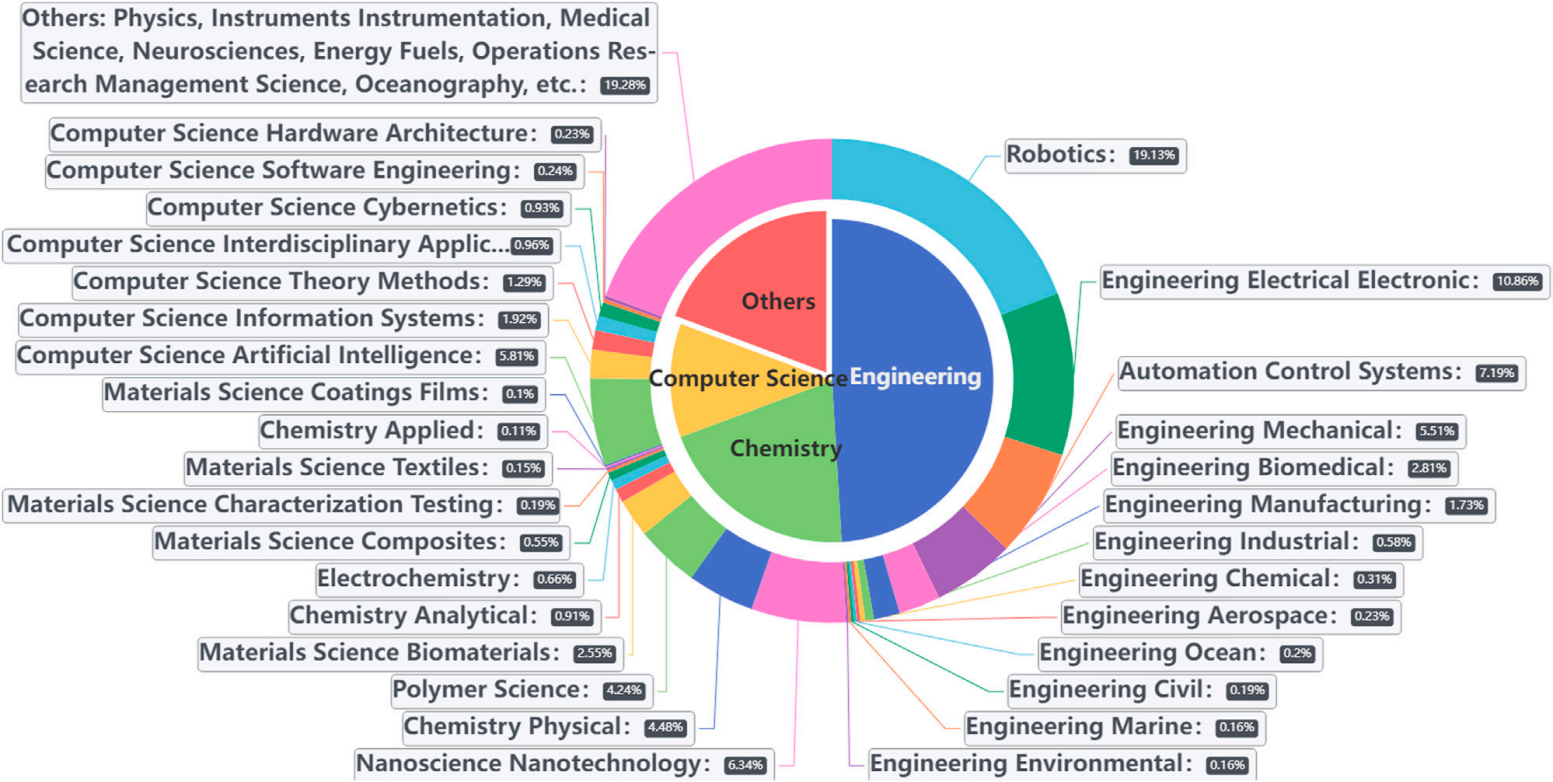
Subject areas and their proportions in soft robotics.

Soft robotics is a interdisciplinary field, where researchers from a number of subject areas have contributed to develop this field. The related subject areas and their proportional contribution in the field are depicted in Figure 3. The areas are assigned by WoS. It is observed that Engineering is the most prominent subject area for soft robotics research, followed by Chemistry, Others, and Computer Science. To be more specific, Robotics (19.13%), Engineering Electrical Electronic (10.86%), and Automation Control Systems (7.19%) are ranked as the top three in the Engineering area. On the other hand, Nanoscience Nanotechnology (6.34%), Chemistry Physical (4.48%), and Polymer Science (4.24%) account for the top three in the Chemistry area. Computer Science Artificial Intelligence (5.81%) is the top one sub-area in the Computer Science area. Futhermore, Others include Physics, Instruments Instrumentation, Medical Science, Neurosciences, Energy Fuels, etc., which suggests that the soft robotics is a highly diverse field involving interdisciplinary subject areas. Based on the main themes of these subject areas, several related research topics can be identified: (1) electronics and control technologies for actuation, sensing, and control, (2) advanced materials and their applications in soft robotics, and (3) artificial intelligence enabling intelligence for soft robotics.

### 3.5 Contribution of Leading Journals

There are a number of journals associated with soft robotics due to the inherent interdisciplinary subject areas involved in soft robotics. Table 3 lists the top 20 journals ranked by the total number of articles and their related impact indicators. It is observed that the majority of the journal’s categories belong to the subject areas and sub-areas in Section 3.4. IEEE Robotics and Automation Letters has published the most articles (377), followed by Soft Robotics (288) and Smart Materials and Structures (249). In terms of impact factor (IF), Advanced Materials (30.254) has the highest score among all journals and Soft Robotics (9.904) leads the list in the Robotics category. Regarding average journal impact factor percentile (AJP), Advanced Materials (97.578%), Nature Communications (95.205%), and Soft Robotics (94.643%) rank as the top three journals. Apart from robotics-oriented journals, the top journals contributing to soft robotics are in the fields of Chemistry and Material Science such as Advanced Materials, Advanced Functional Materials, Soft Matter, Smart Materials and Structures, ACS Applied Materials Interfaces etc., suggesting materials research play a key role in the soft robotics field.

**TABLE 3:** The Top 20 Most productive journals of publications during 2010–2021.

Rank	Journal title	TA	Categories	5 Year IF	AJP
1	IEEE Robotics and Automation Letters	377	Robotics	3.856	69.643
2	Soft Robotics	288	Robotics	9.904	94.643
3	Smart Materials and Structures	249	Instruments *&* Instrumentation; Materials Science, Multidisciplinary	3.893	67.175
4	ACS Applied Materials *&* Interfaces	241	Nanoscience *&* Nanotechnology; Materials Science, Multidisciplinary	9.570	83.928
5	Bioinspiration *&* Biomimetics	178	Robotics; Engineering, Multidisciplinary; Materials Science, Biomaterials	3.520	49.107
6	Advanced Materials	174	Nanoscience *&* Nanotechnology; Chemistry, Physical; Physics, Condensed Matter; Physics, Applied; Materials Science, Multidisciplinary; Chemistry, Multidisciplinary	30.254	97.578
7	Advanced Functional Materials	159	Nanoscience *&* Nanotechnology; Chemistry, Physical; Physics, Condensed Matter; Physics, Applied; Materials Science, Multidisciplinary; Chemistry, Multidisciplinary	18.125	94.608
8	IEEE-ASME Transactions on Mechatronics	121	Engineering, Mechanical; Engineering, Electrical *&* Electronic; Automation *&* Control Systems; Engineering, Manufacturing	5.974	81.736
9	Advanced Materials Technologies	112	Materials Science, Multidisciplinary	7.738	82.239
10	Sensors and Actuators A: Physical	96	Instruments *&* Instrumentation; Engineering, Electrical *&* Electronic	3.134	70.534
11	Soft Matter	91	Physics, Multidisciplinary; Polymer Science; Chemistry, Physical; Materials Science, Multidisciplinary	3.705	63.554
12	IEEE Transactions on Robotics	90	Robotics	7.874	87.500
13	Actuators	82	Engineering, Mechanical; Instruments *&* Instrumentation	2.623	43.151
14	Journal of Bionic Engineering	78	Robotics; Engineering, Multidisciplinary; Materials Science, Biomaterials	3.035	45.618
15	International Journal of Robotics Research	77	Robotics	6.376	76.786
16	Advanced Robotics	70	Robotics	1.637	19.643
17	Lecture Notes in Artificial Intelligence	70	Computer Science, Artificial Intelligence	n/a	12.025
18	Nature Communications	70	Multidisciplinary Sciences	15.805	95.205
19	IEEE Access	65	Engineering, Electrical *&* Electronic; Tele- communications; Computer Science, Information Systems	3.671	62.308
20	International Journal of Advanced Robotic Systems	64	Robotics	1.735	16.071

IF, impact factor; AJP, average journal impact factor percentile (%).

## 4 The Intellectual Structure of Soft Robotics

CiteSpace visualizes the literature as a network constructed from a collection of individual networks. Each network is consisted of articles published over a one-year time interval, namely a time slice. CiteSpace connects these disparate networks to create an overview of how a scientific field has progressed over time. It has been demonstrated that networks formed in this manner capture the underlying scientific community’s research interests (Chen, 2012; Chen et al., 2014; Olawumi and Chan, 2018; Zheng et al., 2020). Here, we use CiteSpace to characterize the intellectual structure and patterns of change in such networks in terms of a variety of visual attributes. Note that the data set analyzed by CiteSpace are references of our WoS collection with 10,504 publications. Therefore, some of the references were published before 2010 and may include other types of publications in addition to articles, proceedings papers, and reviews.

### 4.1 Co-Occurrence Network Analysis

Each research paper usually cites a number of references. In a co-citation network, these references are represented as nodes. The degree of connectivity between the nodes of these references indicates how frequently they are cited by the same articles. The assumption is that if two references are frequently cited together, the two references must be related in some way. The inherent connectivity reflects the comprehensive relationship between the cited references and the articles that cite them.

**FIGURE 4:**
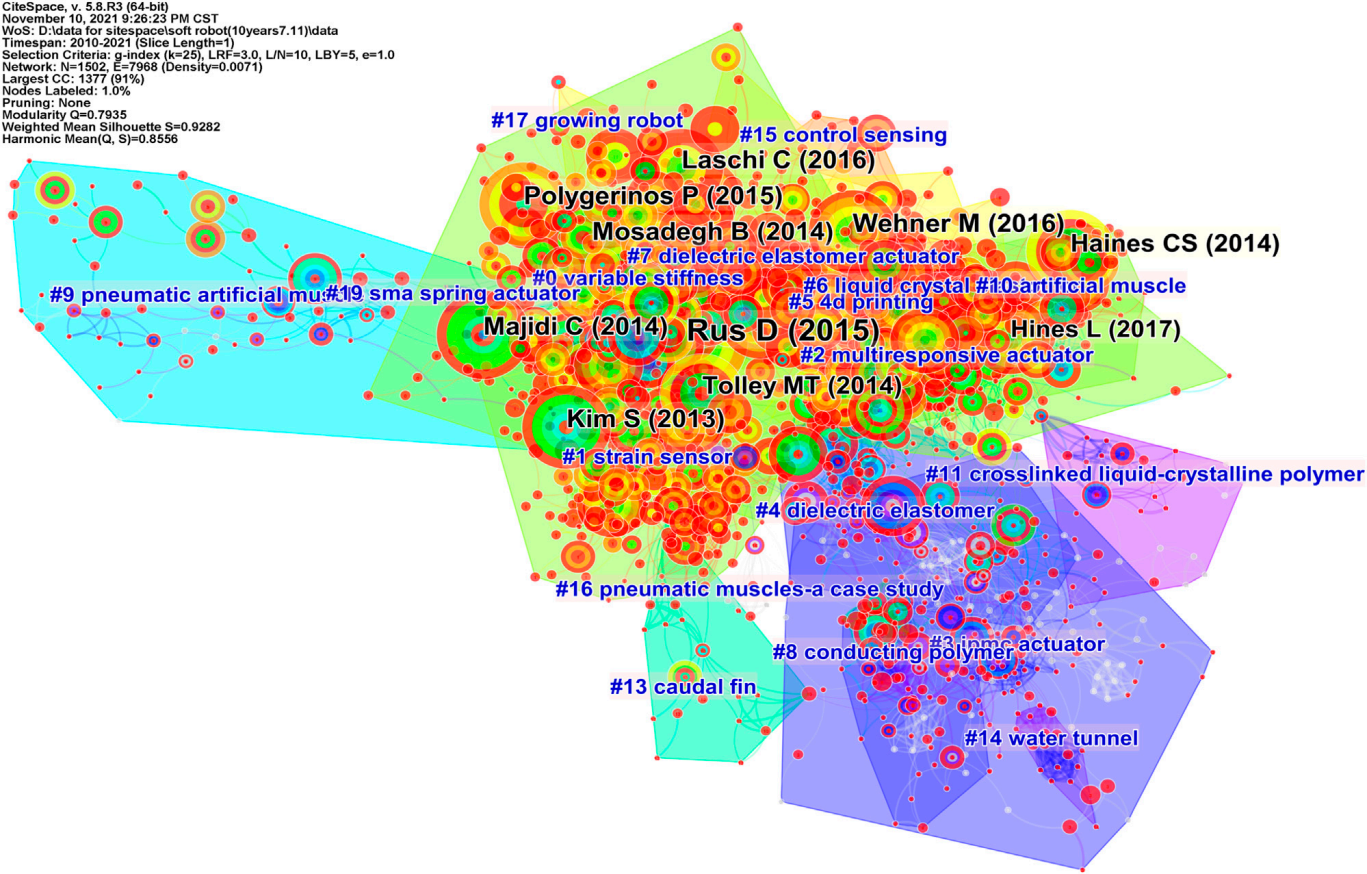
Trajectories of relevant research shown in a network of co-cited references and clustering from titles, where clusters are labeled in blue text and landmark articles in black.

The overview of the co-cited network on soft robotics depicted with CiteSpace is shown in Figure 4. Each node is a reference cited by publications in our WoS collection and is depicted with a series of citation tree-rings across the series of time slices. Note that the nodes are not necessarily in our WoS collection, but the references cited by our WoS collection. The size of a node means the number of citations the associated reference has received: the larger a node, the more citations the node has received. The color of a node’s rings correspond to the time it is cited: the darker blue, the earlier it was cited; and the more red, the more recent it was cited. The thickness of the red rings outside of many nodes indicates how many recent citations that reference has received: the thicker the ring, the more citations; and the more red the ring, the more recent the citations. A red dot at the center of a node corresponds to citation bursts, meaning sudden increases of citations are detected. The color of nodes can be used to trace the timeline of citations and citation bursts are useful to trace the emerging research topics. The connecting curves indicates how often the references are cited together: the thicker the curves, the more often the references are co-cited.

CiteSpace generates 19 clusters based on the association between the nodes, and the areas of different clusters are filled with different colors, as shown in Figure 4. Again, blue and red areas mean earlier and more recent citations, respectively. The clusters are named from titles of citing articles of the cluster using CiteSpace’s built-in algorithm LLR (log-likelihood ratio). Similar to the co-occurrence map, the LLR algorithm also computes based on the number of co-occurring events and is widely used in text computing (Kim and Chen, 2015). The clusters’ ID numbers are sorted by the number of nodes inside: the more nodes inside that cluster, the smaller the ID number. For example, the cluster variable stiffness has the most nodes and is labeled as 0 by CiteSpace.

Table 4 lists the top 10 clusters by their size, that is the number of nodes or members in each cluster. Clusters with few members tend to be less representative than larger clusters because small clusters are likely to be formed by the citing behavior of a small number of publications. The quality of a cluster is also evaluated by the silhouette score (S-score), which represents the cluster’s homogeneity or consistency. Homogeneous clusters tend to have silhouette values close to 1. The silhouette scores of the clusters range from 0.817 (Cluster #2) to 0.987 (Cluster #9), suggesting that our clustered members are highly homogeneous. The average years of publication of the clusters $\left(\bar{Y}\right)$ indicates the cluster’s recentness. For example, Cluster #3 has an average publication year of 2008. The most recently formed cluster in Table 4, Cluster #6 on liquid crystal elastomer, has an average year of 2017.

**TABLE 4:** The top 10 largest clusters.

Cluster #	Label (LLR)	Size	S-score	$\bar{Y}$
0	variable stiffness	288	0.909	2014
1	strain sensor	204	0.946	2015
2	multiresponsive actuator	142	0.817	2015
3	ipmc actuator	119	0.925	2008
4	dielectric elastomer	109	0.971	2009
5	4d printing	86	0.866	2016
6	liquid crystal elastomer	84	0.959	2017
7	dielectric elastomer actuator	72	0.936	2016
8	conducting polymer	68	0.972	2010
9	pneumatic artificial muscle	63	0.987	2010

S-score, silhouette score; $\bar{Y}$ , the average year of publication.

### 4.2 Most Cited Articles

The most cited 10 references by our WoS collection are listed in Table 5 and labeled in black in Figure 4, and the larger the font, the more citations that reference has received. They are considered as landmark articles due to their groundbreaking contributions. Cluster #0 has 8 articles in the top 10 landmark articles, four of which are general reviews of the soft robotics field at that time. Each of Clusters #5 and #10 has one. The most cited article in our dataset is a review article by Rus and Tolley (2015) with 1052 citations. The second one is by Wehner et al. (2016) with 372 citations. They reported a pure soft and untethered robot composed of on-board fuel reservoirs and catalytic reaction chambers. Articles at the third (Kim et al., 2013), fifth (Laschi et al., 2016), and seventh (Majidi, 2014) are all reviews from Cluster #0. The article at the fourth is by Mosadegh et al. (2014), which described a new design for a pneumatic network (pneu-nets) that increases the actuation speed significantly. The sixth article reports a soft robotic glove for combined assistance and at-home rehabilitation (Polygerinos et al., 2015). The article at the eighth presents an untethered soft robot made of pneu-nets (Tolley et al., 2014). The ninth article from Cluster #10 demonstrated artificial muscles made of fishing line and sewing thread (Haines et al., 2014). The article at the 10th is a review from Cluster #5.

**TABLE 5:** The most cited 10 references by our WoS collection.

Cites	References	Cluster #
1052	Rus and Tolley (2015)^a^	0
372	Wehner et al. (2016)	0
316	Kim et al. (2013)^a^	0
303	Mosadegh et al. (2014)	0
300	Laschi et al. (2016)^a^	0
298	Polygerinos et al. (2015)	0
285	Majidi, (2014)^a^	0
271	Tolley et al. (2014)	0
260	Haines et al. (2014)	10
239	Hines et al. (2017)^a^	5

a indicates a review paper.

### 4.3 Cluster Analysis

**FIGURE 5:**
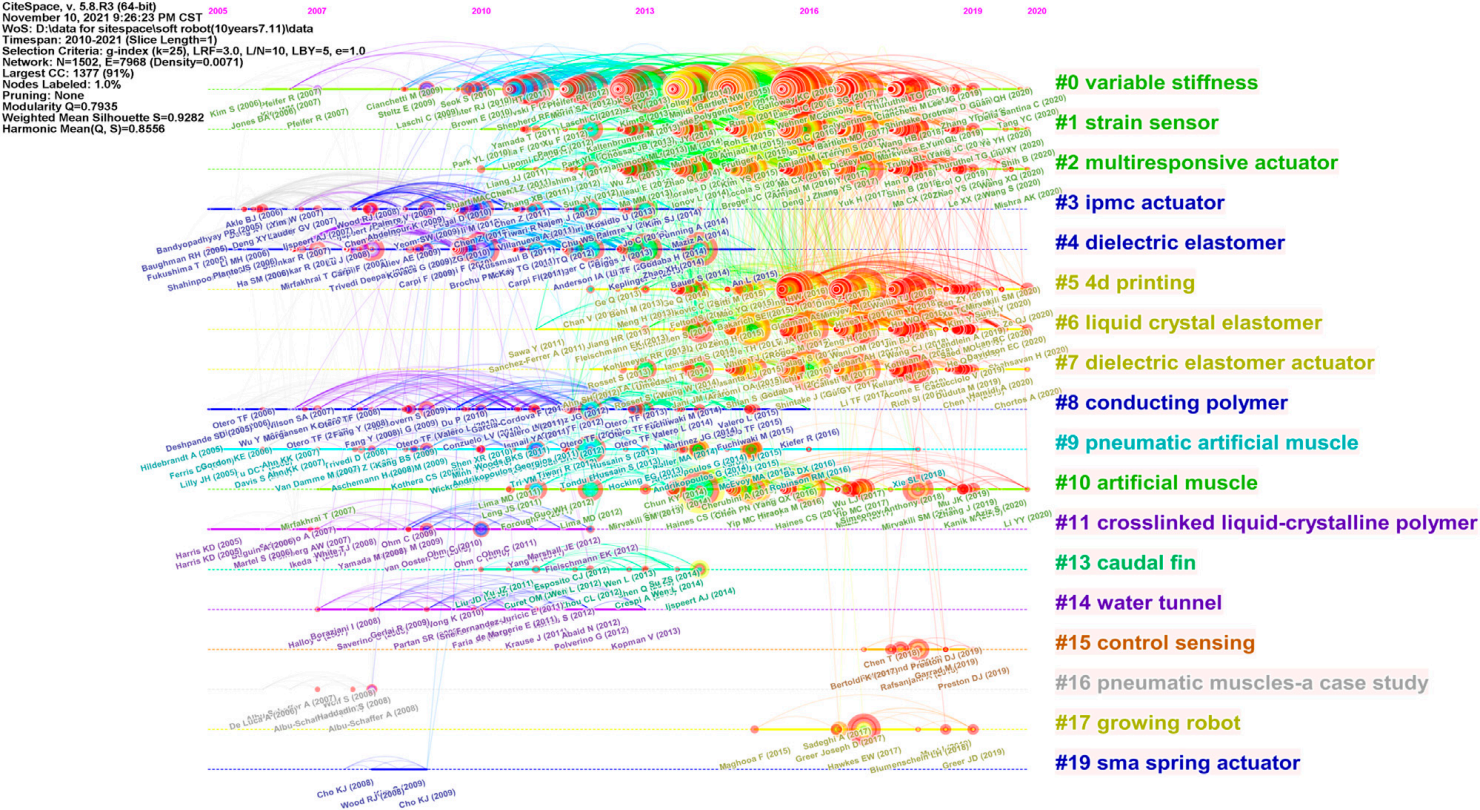
Timelines of co-citation clusters.

Figure 5 visualizes the timeline of distinct co-citation clusters, which is a presentation of Figure 4 by year. It is observed that the highly-cited references in Clusters #3, #4, #8, #9, #11, #13, #14, #16, #19 were mainly published before 2015, meaning they were hot topics earlier. Clusters #15 and #17 spanned during 2017–2019 and 2015–2019, respectively, suggesting they were hot topics a while ago but lacking of evidence for the latest impactful research. Another observation is that Clusters #0, #1, #2, #5, #6, #7, and #10 have been published and cited during the past decade, indicating continuous research interests, and we will focus on analyzing these clusters in the top ten largest clusters, i.e., excluding Cluster #10.

Table 6 lists the most cited 5 references for each of Clusters #0, #1, #2, #5, #6, and #7. Here, cites are the number of citations cited by our WoS collection. It is observed that 1/3 of the highly-cited references are review articles (indicated by a), which is also a sign that the knowledge of soft robotics has been advancing rapidly.

**TABLE 6:** The most cited 5 references for Clusters #0, #1, #2, #5, #6, and #7, respectively.

Cites	References	Cluster #
1052	Rus and Tolley, (2015)^a^	0
372	Wehner et al. (2016)	0
316	Kim et al. (2013)^a^	0
303	Mosadegh et al. (2014)	0
300	Laschi et al. (2016)^a^	0
156	Amjadi et al. (2016)^a^	1
147	Zhao et al. (2016b)	1
131	Larson et al. (2016)	1
100	Dickey, (2017)^a^	1
100	Muth et al. (2014)	1
147	Yuk et al. (2017)	2
67	Breger et al. (2015)	2
64	Taccola et al. (2015)	2
63	Ionov, (2014)^a^	2
62	Morales et al. (2014)	2
239	Hines et al. (2017)^a^	5
207	Gladman et al. (2016)	5
200	Hu et al. (2018)	5
158	Kim et al. (2018)	5
138	Miriyev et al. (2017)	5
129	White and Broer, (2015)^a^	6
128	Palagi et al. (2016)	6
117	Ware et al. (2015)	6
110	Gelebart et al. (2017)	6
99	Wani et al. (2017)	6
157	Shintake et al. (2016)	7
152	Rich et al. (2018)^a^	7
146	Li et al. (2017)	7
132	Acome et al. (2018)	7
85	Gu et al. (2017)^a^	7

a indicates a review paper.

Cluster #0 on variable stiffness includes numerous nodes with red rings of citation bursts indicating a continuous hot topic. Variable stiffness enables high performance and safe human-robot interaction for soft robotics simultaneously. A review by Rus et al. (Rus and Tolley, 2015) is the most cited article both in Cluster #0 and by our WoS collection. This article examines the then developments of soft robotics and major design components, and proposes ideas for the future development including programmable stiffness, proprioceptive sensing, contact modelling etc., which had a profound impact on the soft robotics community. The two regular papers (Mosadegh et al., 2014; Wehner et al., 2016) except for the two review papers (Kim et al., 2013; Laschi et al., 2016) in Clusters #0 focus on pure soft robots and fast soft actuators, respectively. The top cited references in Cluster #0 seems to be not specifically on variable stiffness. It is unclear whether it is due to the limitation of using terms from citing articles to name clusters, or some of them mentioned variable stiffness as an important attribute which inspired roboticists to develop variable-stiffness soft robotics.

The core members of Cluster #1 represent major milestones in relation to strain sensor, notably Zhao et al.’ article on stretchable optical waveguides for strain sensing in a prosthetic hand (Zhao et al., 2016b), Larson et al.’s article on stretchable electroluminescent skin (Larson et al., 2016), and Muth et al.’s article on 3D Printing of strain sensors (Muth et al., 2014). In addition, Amjadi et al. (2016) and Dickey (Dickey, 2017) reviewed stretchable sensors and soft-and-stretchable electronics using liquid metal, respectively. The above indicate a trend for the application and manufacturing of stretchable sensors, which enable potential for exteroception, proprioception, and wearables soft robotics.

Cluster #2 contains further milestones in multiresponsive actuator. Hydrogels, as a sensitive type of polymer capable of producing large, reversible deformations under multiple external stimuli such as temperature, light, and pH, have attracted great attention in the field of soft actuators. The most cited work in this cluster is the 2017 article by Yuk et al. (2017). Their work pioneered the high-speed, high-force, and optically and sonically camouflaged hydrogel actuators. Another prominent member of the cluster is the 2015 article by Breger et al. (2015). Their work reported self-folding thermo-magnetically responsive soft microgrippers and opened up new grounds for multiresponsive soft robots. Morales et al. created the micro tool clamp by coupling photo-cross-linked pNIPAM-AAc soft-hydrogel with a nonswellable and stiff segmented polymer, which relies on water penetration to provide a swelling response to external stimuli (Morales et al., 2014). Other materials have also been explored in addition to hydrogels. Taccola et al. (2015) developed soft actuators controlled by either joule effect or environmental humidity variation using PEDOT:PSS and PDMS. These research also reflect that chemistry and material science play vital roles in soft robotics, which is consistent with our previous findings.

Another observation is that the mean years of the largest 3 Clusters #0, #1, and #2 are around 2015. In addition, around this time, a large number of researchers systematically reviewed the whole field of soft robotics and conducted research from various aspects, which may have helped to define multiple key aspects of soft robotics and accelerate the development of soft robotics. These suggest that these clusters are structurally essential and inspirational. The above may also explain why the soft robotics field started to take off since 2016 in Figure 2.

Cluster #5 on 4D printing, with an average year of 2016, is one of the youngest clusters. 4D printing is a manufacturing method that combines stimuli-responsive matter with 3D printing to create robots that can self-transform into specified shapes in response to external excitation. This technique eliminates the need for molding which was the previous major manufacturing approach and enables rapid prototyping of soft robots. The most cited article in this cluster, Gladman et al. (2016), demonstrated how bionic hydrogel composite can be directly manufactured using 4D ink-jet printing technology. Their work is a major milestone. Another prominent work, Kim et al. (2019), reported direct ink writing of elastomer composites containing ferromagnetic microparticles and suggested new possibilities for applications in flexible electronics, biomedical devices and soft robotics. It is noted that this cluster also includes the publication with the highest citation burst in 2020, which will be further analyzed in Section 5.

Cluster #6 on liquid crystal elastomer (LCEs) is the most recently (2017) formed cluster. The inherent mechanooptic and thermomechanical effects in LCEs allow for both light and heat actuation, enabling multiresponsive actuators and remote powering and control for untethered soft robotics. The one that has the highest citation is a review article by White et al., in 2015 (White and Broer, 2015). It reviewed liquid crystalline polymeric materials with emphasis on the thermally and photogenerated macroscale mechanical responses, which opened up opportunities for mechanical designers to employ these materials in soft robotics. The second most cited work is by Palagi et al. (2016) and they developed a robot with LCEs that can be propelled by structured monochromatic light. In addition, the rest of the 3 highly cited articles all present efficient microactuators made of LCEs (Ware et al., 2015; Gelebart et al., 2017; Wani et al., 2017).

Cluster #7 is also a relatively young cluster with an average year of 2016, indicating that the dielectric elastomer actuator (DEAs) is likely an emerging research topic. DEAs are composed of a dielectric elastomeric film sandwiched between two compliant electrodes. By applying a high voltage across the electrodes, which generates an electrostatic attraction between them, squeezing the elastomer membrane, resulting in elastomer thickness reduction and area expansion. DEAs stands out because of their exceptionally fast response, light weight, and large actuation stroke. The most cited paper in this cluster, Shintake et al. (2016), presented a novel electrode arrangement electrode arrangement for soft grippers to optimize electroadhesion and electrostatic actuation simultaneously, generating over ten times higher electroadhesion force than would be obtained from a conventional DEA electrode configuration. The second most cited article is by Li et al. (2017). They developed a fast-moving soft electronic fish by combining DEAs and ionically conductive hydrogel, resulting in both high speed and consistent performance. Another article by Acome et al. (2018) addressed the problem that traditional DEAs actuators are likely to fail due to electrode breakdown or electrical aging by using self-healing liquid dielectric. The second most cited reference in this cluster is a review article by Rich et al. (2018), where they pointed out that improving DEAs’ output force and stroke will enable further applications in untethered systems. For a detailed review of DEAs actuated soft actuators, readers are encouraged to refer to (Gu et al., 2017).

It is noticed that some of the clusters have similar names but form different clusters, such as clusters #6 (liquid crystal elastomer) and #11 (crosslinked liquid-crystalline polymer). We further investigate the differences between these clusters. The references in cluster #11 focus on the early pioneering studies of applications of liquid crystal elastomers, such as Van Oosten et al. (2009) and Ikeda et al. (2007). Cluster #6 includes the widespread use of liquid crystal elastomers for soft robotics in more recent times. This difference can also be noted from Figure 5. Cluster #9 (pneumatic artificial muscle) includes many references related to pneumatic muscles (e.g., Lin et al. (2015); Sárosi et al. (2015)), while quite a few references in cluster #16 (pneumatic muscles-a case study) involve commercialized robotics, such as Albu-Schäffer et al. (2007a), Albu-Schäffer et al. (2007b)) and it is a relatively older cluster. Cluster #10 (artificial muscle) is concerned with general artificial muscles. A representative article uses polymer fibers, such as fishing line, to create artificial muscles (Haines et al., 2014). Since the clusters are formed by co-citation behavior, it is expected that the clusters can be formed by co-citation at different times, different actuation methods, etc., which differentiates them from similar clusters. In this way, these clusters may include different information on similar fields and trends at different times.

### 4.4 Citation Bursts

Citation bursts are useful to trace research focus. A citation burst has two key aspects: the strength and the duration of the burst. Table 7 lists the top 20 references with the strongest citation bursts across the entire dataset during the period of 2010–2021. It is observed that 19 of the 20 references were published between 2008 and 2014, with the majority of their citation bursts occurring between 2010 and 2018, indicating that these references are pioneering work that had significant impacts on the soft robotics community, especially during the early days. Among the top 20 references with strongest citation bursts, Shepherd et al. (2011), titled “Multigait soft robot,” is on the top of the list with the burst strength of 91.96. It has the strongest citation burst, which started in 2013, the third year after publication, and ended in 2016. This article published in Proceedings of the national academy of sciences proposed the concept of a locomotive quadrupedal soft robot. The second article (Kim et al., 2013) with strongest citation bursts is one of the earliest reviews on soft robotics. The next article is by Ilievski et al. (2011), the author who used pneumatic networks (PneuNets) to create large-amplitude actuations in soft elastomers by pressurizing embedded channels. Notably, almost half of the references focus on various soft actuators (Brochu and Pei, 2010; Ilievski et al., 2011; Anderson et al., 2012; Lima et al., 2012; Martinez et al., 2013; Haines et al., 2014; Mosadegh et al., 2014) and manipulators (Laschi et al., 2012; Martinez et al., 2012). The above suggest that soft actuators have been the core aspect of the soft robotics research. In addition, 5 references were on locomotive robots with focus on locomotion (Lin et al., 2011; Shepherd et al., 2011; Seok et al., 2013; Tolley et al., 2014) and camouflage (Morin et al., 2012), indicating the interests of exploring unpredictable environments with soft robots. The most recent reference with the strongest citation bursts is by Kim et al. (2018), which reported 3D printing of programmed ferromagnetic domains in soft materials that enable fast transformations between complex 3D shapes via magnetic actuation. This work represents two of the emerging trends in soft robotics, ferromagnetic composites and 4D printing, which will be discussed further in Section 5.

**TABLE 7:** The top 20 references with the strongest citation bursts during 2010–2021.

References	Title	Burst	Duration	Range (2010–2021)	Cluster #
Shepherd et al. (2011)	Multigait soft robot	91.96	2013–2016	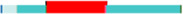	0
Kim et al. (2013)^a^	Soft robotics: a bioinspired evolution in robotics	90.84	2014–2018	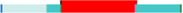	0
Ilievski et al. (2011)	Soft robotics for chemists	74.69	2012–2016	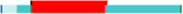	0
Brochu and Pei, (2010)^a^	Advances in dielectric elastomers for actuators and artificial muscles	69.47	2010–2015	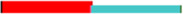	4
Laschi et al. (2012)	Soft robot arm inspired by the octopus	53.58	2012–2017	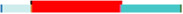	0
Lin et al. (2011)	GoQBot: a caterpillar-inspired soft-bodied rolling robot	48.35	2012–2016	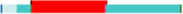	0
Martinez et al. (2013)	Robotic tentacles with three-dimensional mobility based on flexible elastomers	47.45	2014–2018	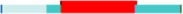	0
Seok et al. (2013)	Meshworm: a peristaltic soft robot with antagonistic nickel titanium coil actuators	42.04	2015–2018	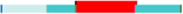	0
Anderson et al. (2012)^a^	Multi-functional dielectric elastomer artificial muscles for soft and smart machines	41.78	2013–2017	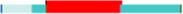	4
Trivedi et al. (2008)^a^	Soft robotics: biological inspiration, state of the art, and future research	40.12	2010–2013	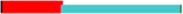	4
Morin et al. (2012)	Camouflage and display for soft machines	37.93	2013–2017	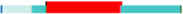	0
Haines et al. (2014)	Artificial muscles from fishing line and sewing thread	36.98	2015–2018	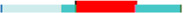	10
Chen et al. (2010)	Modeling of biomimetic robotic fish propelled by an ionic polymer–metal composite caudal fin	34.34	2011–2015	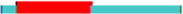	3
Lima et al. (2012)	Electrically, chemically, and photonically powered torsional and tensile actuation of hybrid carbon nanotube yarn muscles	33.21	2014–2017	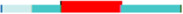	10
Tondu (2012)^a^	Modelling of the McKibben artificial muscle: a review	32.38	2013–2017	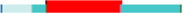	9
Pfeifer et al. (2012)^a^	The challenges ahead for bio-inspired “soft” robotics	30.67	2013–2017	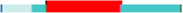	0
Tolley et al. (2014)	A resilient, untethered soft robot	30.01	2015–2018	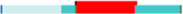	0
Mosadegh et al. (2014)	Pneumatic networks for soft robotics that actuate rapidly	29.43	2015–2019	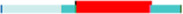	0
Martinez et al. (2012)^a^	Elastomeric origami: programmable paper-elastomer composites as pneumatic actuators	27.37	2014–2017	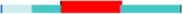	0
Kim et al. (2018)	Printing ferromagnetic domains for untethered fast-transforming soft materials	27.17	2020–2021	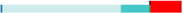	5

a indicates a review paper.

## 5 Emerging Trends

Considering the period from 2010 to 2021, the growth of the number of conferences and academic publications related to soft robotics appears to be substantial, without doubt, the soft robotics has become one of the important topics in the realm of robotics. It is going to attract more and more research efforts, at least, in the following few years. Combining the results of the cluster analysis and citation bursts in Section 4, this section analyzes emerging trends for future research focuses in soft robotics.

References with high strength of citation bursts occurring in the most recent years are considered to represent the emerging trends (Chen et al., 2012). The citation burst suggests that the scholars have paid special attention on the corresponding publications and fields. Table 8 lists the top 5 references with the strongest citation bursts starting in 2018, 2019, and 2020, respectively. In addition, their top 3 citing articles with the highest cites are listed excluding reviews. Table 8 contains 54 articles in total considering the overlap between references and citing articles. For example, Hu et al. (2018) is not only the reference with the strongest citation burst in 2019 but also the top one citing article of Wang et al. (2014).

**TABLE 8:** The top 5 references with the strongest citation bursts starting 2018, 2019, and 2020, respectively.

References	Title	Burst	Duration	Range (2010–2021)	Citing articles
Wang et al. (2014)	Locomotion of inchworm-inspired robot made of smart soft composite	9.6	2018–2019	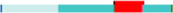	Hu et al. (2018)
					Wang et al. (2018a)
					Gu et al. (2018)
Kim et al. (2014)	Stretchable silicon nanoribbon electronics for skin prosthesis	9.24	2018–2019	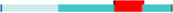	Hua et al. (2017)
					Wang et al. (2018b)
					Lei and Wu, (2018)
Zhao et al. (2016a)	A helping hand: soft orthosis with integrated optical strain sensors and EMG control	8.28	2018–2019	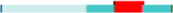	Truby et al. (2018)
					Cappello et al. (2018)
					Meerbeek et al. (2018)
Homberg et al. (2015)	Haptic identification of objects using a modular soft robotic gripper	8.27	2018–2018	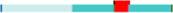	Truby et al. (2018)
					Thuruthel et al. (2019)
					Elgeneidy et al. (2017)
Hong et al. (2015)	3D printing of highly stretchable and tough hydrogels into complex, cellularized structures	7.96	2018–2019	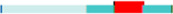	Liu et al. (2018)
					Gao et al. (2018)
					Guo et al. (2020)
Hu et al. (2018)	Small-scale soft-bodied robot with multimodal locomotion	24.48	2019–2021	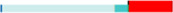	Kim et al. (2019)
					Xu et al. (2019)
					Li et al. (2019)
Shintake et al. (2018)^a^	Soft robotic grippers	24.1	2019–2021	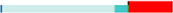	Yan et al. (2020)
					Ze et al. (2019)
					Yang and Yuan, (2019)
Rich et al. (2018)^a^	Untethered soft robotics	18.32	2019–2021	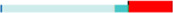	Kim et al. (2019)
					Cui et al. (2019)
					Saed et al. (2018)
Whitesides, (2018)^a^	Soft robotics	15.53	2019–2021	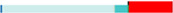	Wang et al. (2019)
					He et al. (2019)
					Xiao et al. (2019)
Polygerinos et al. (2017)^a^	Soft robotics: review of fluid- driven intrinsically soft devices; manufacturing, sensing, control, and applications in human-robot interaction	13.88	2019–2021	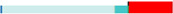	Skylar-Scott et al. (2019)
					Thuruthel et al. (2019)
					Ze et al. (2019)
Kim et al. (2018)	Printing ferromagnetic domains for untethered fast-transforming soft materials	27.17	2020–2021	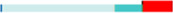	Yuk et al. (2020)
					Sitti and Wiersma, (2020)
					Zhao et al. (2020)
Cianchetti et al. (2018)^a^	Biomedical applications of soft robotics	20.54	2020–2021	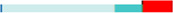	Du et al. (2020)
					Zhang et al. (2020)
					Huang et al. (2020)
Thuruthel et al. (2019)	Soft robot perception using embedded soft sensors and recurrent neural networks	18.73	2020–2021	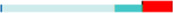	Zhu et al. (2020)
					Jin et al. (2020)
					Lu et al. (2020)
Kim et al. (2019)	Ferromagnetic soft continuum robots	16.82	2020–2021	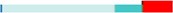	Du et al. (2020)
					Li et al. (2020)
					Han et al. (2020)
Wallin et al. (2018)^a^	3D printing of soft robotic systems	16.37	2020–2021	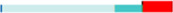	Tan et al. (2019)
					Mishra et al. (2020)
					Huang et al. (2020)

a indicates a review paper.

The top five references by citation burst in 2018 cover a wide range of topics including locomotive robots (Wang et al., 2014), stretchable sensors for skin prosthesis (Kim et al., 2014), soft orthosis hand (Zhao et al., 2016a), object detection with a soft gripper (Homberg et al., 2015), and 3D printing of hydrogels (Hong et al., 2015). It is observed that the burst strength in 2018 is significantly lower than in 2019 and 2020, which could be due to more diverse research interests in 2018. Notably, they were all published before 2017 and none of them have current citation bursts. The references with the most citation bursts in 2019 and 2020 are more recent work. The highest work by this measure in 2019 is a 2018 article in Nature by Hu et al. (2018). They reported magneto-elastic soft millimetre-scale robots that can transit in different liquid and solid terrains, as well as switch between various locomotive modes such as jumping and rolling. In the list of 2020, two articles by Kim et al. (2019) and Kim et al., 2018) reported 3D printing of programmed ferromagnetic domains in soft materials and ferromagnetic soft continuum robots, respectively. They expanded the scope of locomotive soft robots by demonstrating the possibility of 3D printing robots directly, achieving untethered power and control using magnetic fields, as well as navigating through complex and constrained environments. Another notable work is by Thuruthel et al. (2019). Their work reported a neural networks model for characterizing kinematics for a soft continuum actuator. Other articles on the list are review papers with various focuses including soft grippers (Shintake et al., 2018), untethered soft robotics (Rich et al., 2018), fluid-driven soft devices (Polygerinos et al., 2017), soft robotics in general (Whitesides, 2018), biomedical applications of soft robotics (Cianchetti et al., 2018), and 3D printing of soft robotics (Wallin et al., 2018). Compared to the older review articles in Table 7, the variety of review articles in Table 8 is increasing, indicating that soft robotics knowledge is expanding. Based on the references and citing articles in Table 8 and our previous cluster analysis in Section 4, we summarize the following three emerging trends: new materials, 3D or 4D printing, as well as sensing and intelligence.

### 5.1 New Materials

New materials enable new actuation, sensing, control, and applications, as well as require new integration methods, which expands the soft robotics field as a whole. It was found previously that materials related research were critical in soft robotics field: in Section 3.4, Chemistry was found to be the second largest research subarea in soft robotics; and multiple clusters were named after certain materials and materials integration method including Cluster #3, #4, #5, #6, #7, #8, and #11. More importantly, multiple clues have led us to believe that new materials and materials integration are of the most recent emerging trends and potential future directions in soft robotics: the youngest clusters are named after certain materials and integration (Cluster #5, #6, and #7); and the vast majority of the articles in Table 8, which includes both references with recent citation bursts and citing articles with high cites, are related to the development of new materials and composites, as well as materials integration methods.

Multiple materials and composites are gaining momentum. Soft magnetic composites seem to attract the most attention as they have can be controlled by remotely controllable actuating fields and high penetration depths, enabling wireless applications such as drug delivery. These composites are made with various soft polymers embedded with uniformly dispersed magnetized or magnetizable microparticles such as neodymium-iron-boron (NdFeB) particles. These composites often appear as mobile microrobots and are referred to as ferromagnetic soft robots by Kim et al. (2019). Hu et al. (2018) developed a magneto-elastic soft millimetre-scale robot using silicone elastomer embedded with hard NdFeB microparticles, which showed high mobility through multimodal locomotion. Kim et al. (2019) presented self-lubricating soft continuum robots with soft polymer matrix composed of polydimethylsiloxane (PDMS) or thermoplastic polyurethane (TPU), hard NdFeB particles, and a hydrogel skin. It was demonstrated that the robot was able to achieve omnidirectional steering, navigate in a vascular phantom, and deliver laser. Multiple other research efforts have attracted recent attentions by enabling magnetically driven robots with various functional features such as camouflage (Du et al., 2020), sensing and wireless communication (Lu et al., 2020), and shape memory (Ze et al., 2019). A particular interesting work by Li et al. (2020) manipulated droplets by a magnetic-actuated robot made of two steel beads, resulting in a system with blurry boundaries between rigid, fluidic, and soft robots.

Liquid crystal elastomers (LCEs) as a thermally driven actuating material that combines polymer network and liquid crystal mesogens, have also received significant citation bursts. LCEs can be actuated by multiple actuation sources such as direct environmental heating, light through photothermal photochemical effects, or electrical power via combination with resistive heaters, enabling multiresponsive actuators. What many of these recent LCEs related articles have in common appear to be the focus on proposing various techniques of manufacturing and exploring multimodal locomotion. Zhang et al. developed a manufacturing approach for simultaneous welding and aligning LCE materials with different chemical compositions and physical properties by thermal polymerizing prefabricated LCE films with reactive acrylate groups (Zhang et al., 2020). Using this method, multicomponent/multimaterial three-dimensional (3D) LCE robots with multiple 3D geometries were created. Saed et al. (2018) used DIW (direct ink writing) to create molecularly-engineered liquid crystal elastomer actuators. He et al. (2019) created an LCE tubular actuator by sandwiching loosely cross-linked LCE with heating wires, which was then UV irradiated to form a tubular shape. It was demonstrated that by powering the heating wires inside it, multi-directional bending and uniform contraction can be achieved. Xiao et al. reported a soft robots capable of executing various types of biomimetic locomotion, which were also actuated electrically by heating wires (He et al., 2019).

Hydrogels and dielectric elastomers have also gained increased citations. Hydrogels can provide space for ion diffusion transfer and have demonstrated many advantages including ionic conductivity, stretchability, biocompatibility, optical transparency, self-healing capability etc., which makes them good candidates for flexible sensors, conductors, and actuators. Liu et al. designed an anti-freezing hydrogel with zwitterionic poly (ionic liquid) (PIL) for use in a multimodal artificial skin (Liu et al., 2020), which exhibited super stretchability (about 900%), self-healing ability, and high electrical conductivity. Yang et el. developed stretchable and transparent double-network hydrogel ionic conductors which can be used for both sensing and conducting in soft systems (Yang and Yuan, 2019). Mishra et al. reported a soft hydrogel-based actuator that can maintain stable body temperatures via autonomic perspiration (Mishra et al., 2020). Dielectric elastomers have been used for fast actuators and stretchable sensors during the past decade. One particular interesting work by Tan et al. developed a self-healable, low-field illuminating optoelectronic stretchable device by introducing a transparent, high permittivity polymeric dielectric material (Tan et al., 2019).

### 5.2 3D or 4D Printing of Soft Robots

New materials need new materials integration methods. Direct printing attracted the most attention as 13 (24%) articles (Gao et al., 2018; Hong et al., 2015; Huang et al., 2020; Kim et al., 2018; Liu et al., 2018; Saed et al., 2018; Skylar-Scott et al., 2019; Truby et al., 2018; Wallin et al., 2018; Yuk et al., 2020; Zhao et al., 2020; Mishra et al., 2020; Zhang et al., 2020) in Table 8 focus on 3D or 4D printing soft robots, which cover a variety of topics including biomedical devices, drug delivery micro-robots, reconfigurable robots, flexible electronics etc. So-called 4D printing (Tibbits, 2014) combines 3D printing with programmable and stimuli-responsive materials, where the fourth dimension corresponds to a later shape change from the printed structure excited by various external stimuli (heat, electricity, light etc.). The majority of the above publications still use the term 3D printing, indicating the term of 4D printing is not fully differentiated from 3D printing yet. However, the directly printed stimuli-responsive soft robots which later could be controlled by external stimuli already differentiated them from traditional 3D printing as a fact. In the rest of this paper, we will use 3D printing, 4D printing, or direct printing alternatively. In 2018, Wallin et al. (2018) reviewed various 3D-printing materials and printing methods of soft robots such as 3D printing, direct ink writing, fused deposition modelling, inkjet printing, and stereolithography.

3D printing of soft robots provide an excellent, systematic tool to directly fabricate soft robots, which enabled the design of soft robots with sophisticated motion capabilities (jumping, complex 3D movements, gripping and releasing etc.) and integrated sensing. Corresponding to the materials discussed in Section 5.1, 3D printing is used for creating soft robots with ferromagnetic composites (Kim et al., 2018), LCEs (Saed et al., 2018; Zhang et al., 2020), and hydrogels (Hong et al., 2015; Gao et al., 2018; Huang et al., 2020; Mishra et al., 2020). Different materials require different printing methods. Several other materials have also attracted much attention given the variety of materials used in soft robotics. Yuk et al. (2020) 3D printed conducting polymer based on PEDOT:PSS, and further fabricated a soft neural probe capable of *in vivo* single-unit recording. Zhao et al. (2020) developed a direct ink writing protocol to create miniaturized silica aerogel objects. A particular interesting work by Liu et al. (2018) developed a new method and material system capable of 3D-printing living responsive materials and devices. Furthermore, multiple nozzles can be used to deposit different polymers or inks, leading to multi-material prints. For example, a millipede-like soft robot that locomotes by co-printing multiple epoxy and silicone elastomers (Skylar-Scott et al., 2019), and fabrication of soft somatosensitive actuator innervated with multiple soft sensors (Truby et al., 2018). In summary, a series of recent articles have developed various 3D printing systems for a variety of stimuli-responsive soft materials, and multi-material printing have showed the potential for direct printing of soft robots with complex architectures, multimodal motion, and embedded sensing.

### 5.3 Sensing and Intelligence

Soft systems, in contrast to conventional stiff robots, and other piecewise rigid systems with finite degrees of freedom, are continuous elastic bodies with infinite degrees of freedom. This poses new challenges in sensing and proprioception, feedback and adaptive control, path planning, and robot intelligence, all of which are beyond the scope of conventional robotic algorithms. Furthermore, the development of new materials, actuators, and integration methods will require new sensing and intelligence methods in the mean time. Multiple evidences have indicated the emerging trends for sensing and intelligence: two clusters are directly named after sensing including Cluster #1 and #15 on strain sensor and control sensing, respectively, where Cluster #1 is also the second largest cluster and a relatively young one with the average year of 2015. In addition to multiple publications related to sensing discussed previously, 15 articles (about 28%) in Table 8 focus directly on sensing and intelligence of soft robots.

Multimodal stretchable sensing skins have attracted significant attention lately, which are mainly composed of various highly-stretchable conductive polymers and structures. An article by Kim et al. (2014) received widespread attention and citation bursts during 2018–2019. They developed a highly integrated stretchable artificial skin using ultra-thin single crystal silicon nanoribbons (SiNR) sensor arrays and humidity sensors capable of multimodal sensing including strain, press, temperature, and humidity. External stimuli were demonstrated to transfer via prosthesis to specific nerves in human bodies, mimicking real skins. Lei et al. fabricated a supramolecular biomimetic skin using supramolecular polyelectrolyte hydrogels capable of detecting temperature, strain, and stress (Lei and Wu, 2018). Hua et al. created a highly stretchable and adhesive matrix network (SCMN) artificial skin that can detect temperature, intra-surface strain, relative humidity, UV rays, magnetic fields, pressures, and proximity, enhancing multimodal sensing in significantly (Hua et al., 2017). Wang et al. (2019) fabricated a self-healable ternary polymer composite to detect strain and pressure. Han et al. (2020) developed catheter-integrated soft multilayer electronic arrays for multiplexed sensing and actuation during cardiac surgery, which can detect temperature, pressure and electrophysiological parameters.

Multiple other sensors have received increasing attention as well. Li et al. proposed a superhydrophobic paper-based strain sensor which could detect ultra-low strain as low as 0.1% and showed potential for application in wearable devices (Li et al., 2019). Wang et al. developed a compliant ultrathin sensing and actuating electronics innervated fully soft robots with sensors made of single-crystal Si optoelectronic photodetectors, which can sense the environment and perform soft bodied crawling adaptively (Wang C. et al., 2018). Jin et al. demonstrated triboelectric nanogenerator sensors to capture the continuous motion and tactile information for soft gripper (Jin et al., 2020).

It is observed an emerging trend of combining sensing data with data-driven learning algorithms to process complex information such as robots’ own status like shape, and interface information like force. Some of these sensors are even commercially available ones. Homberg et al. (2015) were one of the earliest to adopt learning algorithms in haptic identification of objects with a soft gripper, where commercial resistive flex sensors were used. Elgeneidy et al. (2017) derived both neural network and regression models based on experimental sensing data, and the resulting empirical model was effectively used in closed-loop control. Thuruthel et al. (2019) developed a long short-term memory (LSTM) network with redundant and unstructured sensor topology embedded in soft actuators, which was used for kinematics and force modeling of soft actuators in real time. Zhu et al. (2020) created a tactile feedback smart glove with a triboelectric tactile sensor, and trained a convolutional neural network (CNN) model for object recognition. Meerbeek et al. (2018) reported soft optoelectronic sensory foams with proprioception using multiple machine learning algorithms, where the magnitude and type of deformation for soft actuators can be identified.

In summary, the sensing modality is expanding and approaching human sensations including pressure, vibration, pain, humidity, temperature, etc., which are joint sensing results of various stimuli. Soft roboticists are trying to detect various stimuli accurately, improve sensitivity, and understand various information in order to let robots adapt to and make smart decisions in complex environments.

## 6 Conclusion

In conclusion, the analysis of the literature of soft robotics and a citation-based expansion has outlined the evolutionary trajectory of the collective knowledge over about the last decade and highlighted the areas of active pursuit. We have analyzed the patterns of soft robotics publications, geographic distribution, source journals, source institutions, international collaboration, and main areas. China, USA, Japan, UK, and South Korea are the most productive countries/regions. The most productive countries/regions are located in East Asia, North America, and West Europe. Chinese Academy of Sciences from (China), University of California System, and Harvard University are listed on the top three places according to the number of publications. International collaborations research turn out to generate higher impact. In reference to the journals, IEEE Robotics and Automation Letters ranks the most productive, whereas Soft Robotics holds the top position in journals categorized with “Robotics” in terms of 5 years impact factor. The growth of soft robotics originates from combined efforts of multiple areas such as computer science, chemistry, engineering etc.

Literature clusters and emerging trends identified in the analysis are based on computational properties selected by CiteSpace, which facilitates data-driven analysis of scientific frontiers based on relevant domain literature. The number of review articles on relevant topics is rapidly increasing, indicating the knowledge of soft robotics has been growing rapidly. The visual analysis of the broader domain outlines the major milestones throughout the extensive period of 2010–2021. Several indicators and observations converge to the critical and active emerging trends of new materials, 4D printing, sensing, and intelligence. New materials such as ferromagnetic composites, LCEs, and hydrogels enable new developments in structures, actuation methods, sensing, control, and applications, which are at the forefront of the growth of soft robotics. 4D printing accelerates the manufacturing of soft robots considerably. The development of highly-stretchable sensors and multimodal sensors will dramatically improve soft robots’ somatosensory system and perception of their surroundings. Artificial intelligence will generate profound progress on modeling, control, and sensation for soft robots. These major trends identified in this review have distinct research agendas as well as combined efforts. In a long run, more trends are expected to emerge from other perspectives and existing trends may be accommodated by new levels of integration.
